# Bevacizumab Arrests Osteoarthritis Progression in a Rabbit Model: A Dose-Escalation Study

**DOI:** 10.3390/jcm10132825

**Published:** 2021-06-26

**Authors:** Gianluca Vadalà, Luca Ambrosio, Caterina Cattani, Roberta Bernardini, Antonino Giacalone, Rocco Papalia, Vincenzo Denaro

**Affiliations:** 1 Department of Orthopaedic and Trauma Surgery, Campus Bio-Medico University of Rome, 00128 Rome, Italy; l.ambrosio@unicampus.it (L.A.); r.papalia@unicampus.it (R.P.); denaro@unicampus.it (V.D.); 2 National Institute for Health, Migration and Poverty (NIHMP), 00153 Rome, Italy; cattanicaterina@libero.it; 3 Interdepartmental Service Center—Station for Animal Technology (STA), University of Rome Tor Vergata, 00133 Rome, Italy; roberta.bernardini@uniroma2.it; 4 IRCCS Istituto Ortopedico Galeazzi, University of Milan, 20161 Milan, Italy; antonino.giacalone14@gmail.com

**Keywords:** osteoarthritis, bevacizumab, cartilage, vascular endothelial growth factor (VEGF), neoangiogenesis

## Abstract

Cartilage neoangiogenesis holds a prominent role in osteoarthritis (OA) pathogenesis. This study aimed to assess the efficacy bevacizumab, an antibody against vascular endothelial growth factor and inhibitor of angiogenesis, in a rabbit OA model. Animals were divided into four groups: one receiving a sham intra-articular knee injection and three groups undergoing 5, 10, and 20 mg intra-articular bevacizumab injections. The effect of the antibody on articular cartilage and synovium was assessed through histology and quantified with the Osteoarthritis Research Society International (OARSI) scores. Immunohistochemistry was performed to investigate type 2 collagen, aggrecan, and matrix metalloproteinase 13 (MMP-13) expression. Bevacizumab treatment led to a significant reduction of cartilage degeneration and synovial OA changes. Immunohistochemistry revealed significantly lower cartilage MMP-13 expression levels in all experimental groups, with the one receiving 20 mg bevacizumab showing the lowest. The antibody also resulted in increased production of aggrecan and type 2 collagen after administration of 5, 10, and 20 mg. The group treated with 20 mg showed the highest levels of type 2 collagen, while aggrecan content was even higher than in the healthy cartilage. Intra-articular bevacizumab has been demonstrated to effectively arrest OA progression in our model, with 20 mg being the most efficacious dose.

## 1. Introduction

Osteoarthritis (OA) is a degenerative joint disorder affecting more than 10% of individuals older than 60 years of age. It is characterized by escalating joint pain and stiffness progressively leading to disability, with a significant burden on patients’ overall functionality and quality of life, as well as on healthcare expenditure [1]. Main risk factors comprise aging, female gender, genetics, and alterations of joint biomechanics. Moreover, recent research has outlined the possible role of systemic factors, such as proinflammatory adipokines in obese and diabetic individuals [2] as well as alterations of the gut microbiota in patients with intestinal dysbiosis [3], in the development of OA.

Major pathologic characteristics include articular cartilage damage and thinning, which are associated with chondrocyte hypertrophy, tissue inflammation [4], and extracellular matrix (ECM) disruption [5]. Furthermore, during the osteoarthritic process, new blood vessels originating from the subchondral bone invade the articular cartilage, which is physiologically avascular. Articular cartilage vascular ingrowth has been demonstrated to foster tissue calcification, periarticular osteophyte formation, and sprouting of sympathetic sensory nerve fibers across the tidemark, which seems to significantly contribute to osteoarthritic articular pain [6].

Cartilage neoangiogenesis and nerve growth in OA have been associated with elevated levels of vascular endothelial growth factor-A (VEGF-A, classically referred to as VEGF), a potent proangiogenic factor mainly produced by the synovium and osteoarthritic chondrocytes [7]. Indeed, VEGF synovial levels in patients with OA have been shown to positively correlate with both OA severity [8] and intensity of articular pain [9]. An in vivo study from Ludin et al. [10] demonstrated that intra-articular knee injection of VEGF induced OA-like alterations including cartilage degradation with proteoglycan loss, subchondral bone sclerosis, osteophyte formation, and synovial hyperplasia. Moreover, VEGF signaling in chondrocytes has been associated with several catabolic alterations including increased production of matrix metalloproteinases (MMP)-1, -3, -9, -13, a disintegrin and metalloproteinase with thrombospondin motifs (ADAMTS)-5, nitric oxide (NO), reactive oxygen species (ROS), interleukin (IL)-1β, -6 and tumor necrosis factor (TNF)-α [7]. 

Considering its several implications in OA pathogenesis [11], VEGF has been considered to be a possible target for OA pharmacologic treatment. Bevacizumab is an anti-VEGF monoclonal antibody currently employed in the treatment of solid cancers, age-related macular degeneration, and diabetic retinopathy. In a study from Nagai et al. [12], intravenous and intra-articular administration of bevacizumab were tested in an OA rabbit model. The systemic administration of the antibody resulted in the enhanced gene expression of type 2 collagen and aggrecan in the articular cartilage, while MMP-13 and ADAMTS-5 were significantly less expressed in the synovium. Moreover, both macroscopically and histologically, animals treated with intravenous bevacizumab showed reduced articular cartilage degeneration and osteophyte formation. On the other hand, intra-articular administration of the antibody resulted in better pain control and reduced cartilage degradation compared to both control and intravenous bevacizumab groups. Although bevacizumab has been shown to reduce OA degenerative changes in vivo, the optimal dose and the ideal route of delivery are still topics of debate. In a recent study conducted by Li and colleagues, intra-articular administration of bevacizumab in a rabbit knee immobilization model of OA was investigated and compared with both hyaluronan and triamcinolone acetonide injections. The group treated with bevacizumab demonstrated the lowest degrees of cartilage degradation, synovial hyperplasia, and VEGF- and MMP-1-positive cells [13].

In the present study, we evaluated the effect of intra-articular administration of bevacizumab in a rabbit anterior cruciate ligament transection (ACLT) OA model. Our primary objective was to establish an ideal dose for the intra-articular administration of bevacizumab. Therefore, different doses of bevacizumab were tested, and cartilage and synovial morphology, type 2 collagen, aggrecan, and MMP-13 expression were assessed.

## 2. Materials and Methods

### 2.1. Experimental Animals

All experimental procedures involving animals were approved on 21 March 2018 by the local animal facility in conformity with the guidelines for animal maintenance. The study protocol was approved by the Italian Ministry of Health under the name “Modulazione dell’angiogenesi nel trattamento dell’osteoartrosi” according to the law 116/1992 (approval date: 3 October 2012). All experiments were performed according to the Animal Research: Reporting of In Vivo Experiments (ARRIVE) guidelines 2.0 [14]. In total, 24 male New Zealand white rabbits aged between 5 and 6 months with an average weight of 5 ± 0.5 kg were obtained from a licensed vendor (Harlan Laboratories, Udine, Italy) and utilized in this study. The animals were kept in standard cages, one per cage, at a controlled temperature (20 ± 2 °C) with a relative humidity between 50% and 55% and were fed with standard diets. All animals were randomly allocated to their cage and bred under the same conditions.

### 2.2. Surgical Procedure

OA was induced following ACLT, which has been shown to rapidly prompt extensive structural changes within the joint, thus being an ideal model for pharmacologic studies [15,16]. Unilateral ACLT was performed under anesthesia induced by an intravenous injection of sodium pentobarbital (30 mg/Kg). In brief, one knee was shaved and sterilized with betadine solution, and a medial parapatellar incision was performed. The patella was dislocated laterally, and the knee was placed in full flexion. The ACL was then individuated and transected with a sharp blade. The joint capsule was irrigated with sterile saline solution and closed along with the synovial membrane using absorbable Vicryl 4-0 sutures. The overlying skin was sutured with nonabsorbable Nylon 3-0 sutures. All animals were allowed to freely walk in their cages after the procedure. All surgical procedures were carried out by the same surgeon.

### 2.3. Knee Injection

After the surgical procedure, the animals were randomly divided into 4 groups: a control group (*n* = 6) receiving an intra-articular injection of 0.8 mL sterile saline solution (NT) and the others undergoing a knee injection of bevacizumab (Avastin^©^ 100 mg/4 mL, Roche, Basel, Switzerland) 5 (*n* = 6), 10 (*n* = 6), and 20 mg (*n* = 6) in the same volume (0.8 mL). Random numbers were generated using the standard = RAND() function in Microsoft Excel. All the injections were performed using a 27 G needle inserted through the lateral infrapatellar area toward the intercondylar space of the femur in a deep knee-flexed position. The first injection was given after 4 weeks after surgery. All animals of each group were treated once in a week over a period of 4 weeks, and they were sacrificed at 3 months from the ACLT surgery by an overdose of sodium pentobarbital. In order to minimize potential confounders, testing order was randomized weekly, with each animal tested at a different time each test day. All individuals involved in the experiments were blinded with regards to group allocation except for those deputed to animal care. No adverse reaction was noted during the treatment. The knee joints were then harvested and underwent histology and immunohistochemistry evaluations. Contralateral intact knees were used as healthy controls. Animals were included in the study if they successfully showed OA changes within the knee joints (e.g., fissures and erosion of cartilage, loss of Safranin O staining, chondrocyte loss, and chondrocyte clustering). 

### 2.4. Histological Evaluation

**Figure 1:**
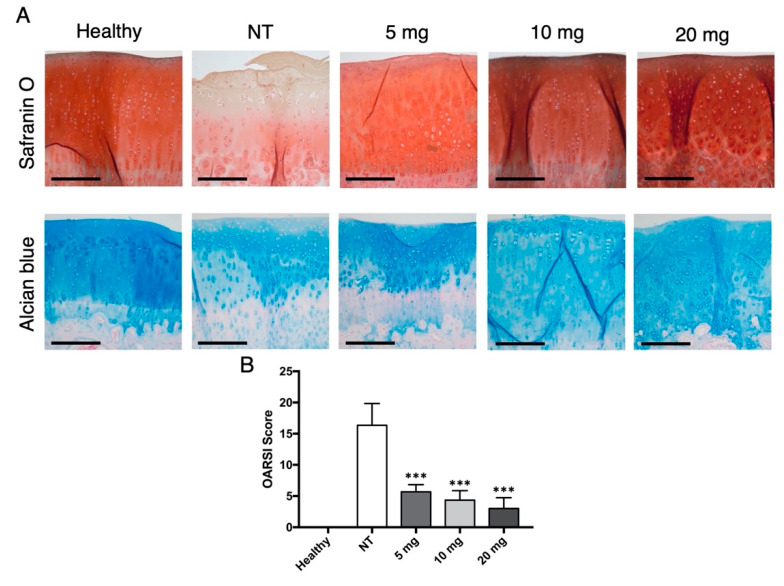
Histologic evaluation of bevacizumab on articular cartilage morphology. (**A**) Safranin O and Alcian blue staining of articular cartilage. The NT group showed the most evident degenerative changes, with reduced staining intensity, surface disruption, and chondrocyte loss. Otherwise, groups treated with bevacizumab displayed fewer osteoarthritic alterations. Scale bars = 200 μm. (**B**) The OARSI score calculated for the Safranin O–fast green cartilage sections confirmed these findings, with significantly decreased values for the groups injected with bevacizumab. *** *p* < 0.001, bevacizumab 5, 10, and 20 mg compared to the NT group. Data are expressed as mean ± SD. NT = not treated.

**Figure 2:**
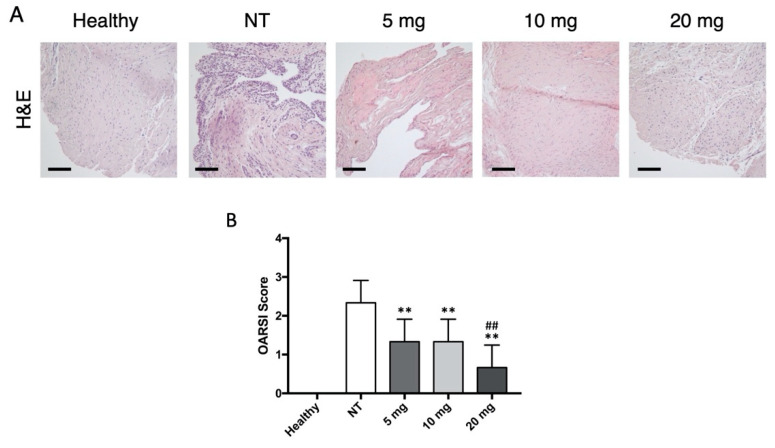
Histologic evaluation of bevacizumab on the synovium. (**A**) H&E staining of synovial fragments showed signs of hyperplasia, vessel proliferation, and inflammatory infiltration in the NT group. Conversely, groups treated with bevacizumab demonstrated substantially fewer alterations. Scale bars = 200 μm. (**B**) The OARSI scores calculated for the H&E synovial sections reflected these findings for each dose, with 20 mg being the most effective in reducing synovial OA changes. ** *p* < 0.01, bevacizumab 5, 10, and 20 mg compared to the NT group. ^##^ *p* < 0.01, bevacizumab 20 mg compared to 5 and 10 mg. Data are expressed as mean ± SD. NT = not treated.

The distal parts of the femur were harvested and fixed with 10% formalin for 24 h. Each specimen was decalcified in a 10% ethylenediaminetetraacetic acid (EDTA) solution in distilled water (pH 7.2) for 4 weeks. Each knee sample was cut into 4 regions from the medial condyle to the lateral condyle, and then the samples were dehydrated in graded alcohols, cleared with xylene, and embedded in paraffin. The samples were sagittally sectioned (5 μm-thick) and then stained with Safranin O/fast green for cellular distribution and ECM composition, Alcian blue for glycosaminoglycans, or used for immunohistochemistry. The synovium was harvested from the infrapatellar fat pad region. Synovial tissue sections were stained with hematoxylin-eosin (H&E) to assess tissue cellularity. Each section was analyzed in a blinded manner by three pathologists who were unaware of the treatment group assignment and quantified using the semiquantitative histological grading system recommended by Osteoarthritis Research Society International (OARSI). The OARSI grading system for cartilage is composed of 4 histological parameters (Safranin O-fast green staining, structure, chondrocyte density, and cluster formation), with a total score ranging from 1 (normal articular cartilage) to 24 points (completely damaged osteoarthritic cartilage). The OARSI grading system for the synovium is based on the evaluation of synoviocyte proliferation and hypertrophy, inflammatory infiltrate composition (granulocytic, fibrinous, and lymphoplasmacytic), and synovial stroma composition (presence of villous hyperplasia, proliferation of fibroblasts/fibrocytes, proliferation of blood vessels, cartilage/bone detritus, and hemosiderosis). The total score ranges from 0 (normal synovium) to 30 points (osteoarthritic synovium) [16]. Histological sections were visualized using an Olympus BX51 (Olympus, Tokyo, Japan) by three independent observers with at least 5 years of experience in the field of musculoskeletal microscopic anatomy. Outcome assessors were not informed on the research question as well as on group allocation. Furthermore, scores were assessed at different timepoints so that observers did not meet and influence each other. At least five sections per each sample were analyzed. Representative images are shown in Figure 1 and Figure 2.

### 2.5. Immunohistochemistry

**Figure 3:**
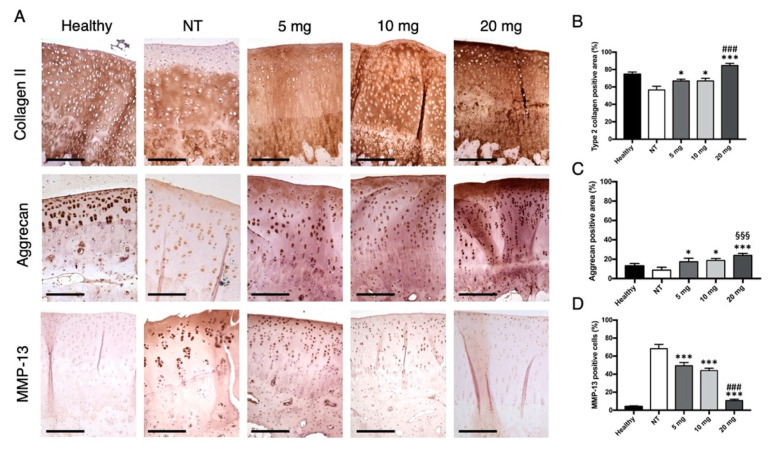
Immunohistochemical evaluation of type 2 collagen, aggrecan, and MMP-13 expression in articular cartilage. (**A**) Representative images showing the immunostaining of type 2 collagen, aggrecan, and MMP-13 in cartilage sections of the four groups. Scale bars = 200 μm. (**B**) Semiquantitative analysis of type 2 collagen-positive area in the articular cartilage of the four groups. The results showed a significant increase in type 2 collagen expression in all animals treated with bevacizumab, with the highest levels in the 20 mg group. (**C**) Semiquantitative analysis of aggrecan-positive area in the articular cartilage of the four groups. The results showed a significant increase in aggrecan expression in all animals treated with bevacizumab, with aggrecan levels in the 20 mg group being even higher than in the healthy control group. (**D**) Semiquantitative analysis of MMP-13-positive cells in the articular cartilage of the four groups. The results showed a significant decrease in MMP-13 expression in all animals treated with bevacizumab, with the lowest levels in the 20 mg group. * *p* < 0.05, bevacizumab 5, 10, and 20 mg compared to the NT group. *** *p* < 0.001, bevacizumab 5 mg, 10 mg and 20 compared to the NT group. ^###^ *p* < 0.001, bevacizumab 20 mg compared to 5 and 10 mg. ^§§§^ *p* < 0.01, bevacizumab 20 mg compared to the healthy control. Data are expressed as mean ± SD. NT = not treated.

Sections were deparaffinized, rehydrated, and then treated in 0.3% H_2_O_2_ (Sigma, St. Louis, MO, USA) for 10 min and washed with phosphate-buffered solution (PBS) for 15 min. For antigen retrieval, sections were either treated with proteinase 0.6 U/mL (Qiagen, Hilden, Germany) or hyaluronidase 1.5 U/mL (Sigma) or chondroitinase ABC 0.25 U/mL (Sigma) for 30 min at 37 °C. Sections were blocked with 1% bovine serum albumin (BSA) in PBS for 30 min and then incubated with the primary antibody. For collagen type II, primary mouse monoclonal antibody (Acris Antibodies, San Diego, CA, USA) was diluted 1:50 in blocking solution for 60 min. For MMP-13, primary mouse monoclonal (Chemicon International, Temecula, CA, USA) was diluted 1:20 in blocking solution and was placed on the section overnight at 4 °C. For aggrecan, primary mouse monoclonal (Novus Biologicals, Littleton, CO, USA) was diluted 1:10 in blocking solution for 2 h. Then, slides were washed with PBS and incubated with a secondary goat antimouse antibody (EnVision^©^+ system-HRP; Dako, Glostrup, Denmark) for 30 min and later soaked with a 3,3′-diaminobenzidine tetrahydrochloride (DAB) solution for 5 min. Finally, the sections were counterstained with Mayer’s hematoxylin for 5 min, dehydrated, and covered. For negative controls, incubation was carried out by a nonspecific IgG antibody at the same concentration as the primary antibody. The staining was evaluated using light microscopy Olympus BX51 (Olympus, Tokyo, Japan). At least five sections per each sample were examined. For each section, five fields were randomly selected and analyzed. The brown area indicated positive staining. The percentage of MMP-13-positive cells was counted, and the mean value was calculated. The expression levels of MMP-13 were expressed as the percentage of positive cells in the total number of cells, with the maximum score being 100%. Similarly, the expression levels of type II collagen and aggrecan in the cartilage were estimated by measuring the ratio between the number of chondrocytes and ECM positively stained compared to the whole matrix. Sections were scored by three individuals under blinded conditions. Representative images are shown in Figure 3.

### 2.6. Statistical Analysis

Data are expressed as mean ± standard deviation (SD) and mean of difference (MD) with 95% confidence intervals (CI). Analysis was performed with Prism 7 (GraphPad Software). Comparisons between the groups were evaluated by one-way ANOVA test with Tukey’s multiple comparisons test. A level of *p* < 0.05 was considered statistically significant. The number of samples for each experimental group was calculated on the basis of the “Resource Equation”: N = (E × T) − T; (10 ≤ E ≤20), where E is the number of samples for each group and T is the number of the experimental groups. In our study, sample size calculated using this method was 24, which is considered adequate to obtain significant results according to our study design [17]. No animal, experimental unit, or data point were excluded from the analysis.

## 3. Results

### 3.1. Histological Examination

Histological assessment of articular cartilage demonstrated a loss of Safranin O and Alcian blue-positive staining in the OA group, with evident fissures and erosions, multifocal loss of chondrocytes and presence of cell clusters. Conversely, the treatment groups showed good staining retention with smooth and regular articular surfaces and no relevant changes in cell density or disposition (Figure 1A). The total OARSI score for articular cartilage (Figure 1B) demonstrated a statistically significant difference between the OA group and all treatment groups (NT vs. 5 mg: MD 10.67, 95% CI from 5.428 to 15.9, *p* < 0.001; NT vs. 10 mg: MD 12, 95% CI from 6.762 to 17.24, *p* < 0.001; NT vs. 20 mg: MD 13.3, 95% CI from 8.095 to 18.57; *p* < 0.001). Although cartilage structure and cell numerosity seemed to progressively improve with bevacizumab dosage, no significant differences were encountered among the three groups (5 vs. 10 mg: MD 1.333, 95% CI from −3.905 to 6.572; 5 vs. 20 mg: MD 2.667, 95% CI from −2.572 to 7.905; 10 vs. 20 mg: MD 1.333, 95% CI from −3.905 to 6.572). 

H&E staining of the synovium showed synoviocyte hyperplasia, vessel proliferation, and lymphoplasmacytic infiltrates in the OA group (Figure 2A). Conversely, tissues treated with bevacizumab displayed a significantly reduced degree of synovial inflammation and OA changes (NT vs. 5 mg: MD 1, 95% CI from −0.3876 to 2.388, *p* < 0.01; NT vs. 10 mg: MD 1, 95% CI from −0.3876 to 2.388, *p* < 0.01; NT vs. 20 mg: MD 1.667, 95% CI from 0.279 to 3.054; *p* < 0.01; Figure 2B). No significant differences were noticed among the treatment groups, except for the group treated with 20 mg bevacizumab (5 vs. 10 mg: MD 0, 95% CI from −1.388 to 1.388; 5 vs. 20 mg: MD 0.667, 95% CI from −0.721 to 2.054, *p* < 0.01; 10 vs. 20 mg: MD 0.6667, 95% CI from −0.721 to 2.054, *p* < 0.01).

### 3.2. Type 2 Collagen, Aggrecan, and MMP-13 Expression in Articular Cartilage

Type 2 collagen expression (Figure 3A) was significantly higher in articular cartilage of animals treated with intra-articular bevacizumab compared to not treated (NT) littermates (NT vs. 5 mg, MD −10.33, 95% CI from −18.21 to −2.453, *p* = 0.0104; NT vs. 10 mg, MD −10 33, 95% from CI −18.21 to −2.453, *p* = 0.0104; NT vs. 20 mg, MD −28, 95% from CI −35.88 to −20.12, *p* < 0.001; Figure 3B). While no significant differences were found between 5 mg and 10 mg (MD 0, 95% from CI −7.88 to 7.88), the administration of 20 mg bevacizumab was demonstrated to increase collagen production more relevantly compared to minor doses (5 vs. 20 mg, MD −17.67, 95% from CI −25.55 to −9.786, *p* < 0.001; 10 vs. 20 mg, MD −17.67, 95% from CI −25.55 to −9.786, *p* < 0.001). Similarly, aggrecan expression was more pronounced in groups treated with bevacizumab (NT vs. 5 mg, MD–8.667, 95% CI from −16.17 to −1.162, *p* = 0.0227; NT vs. 10 mg, MD −10, 95% from CI −17.5 to −2.495, *p* = 0.0191; NT vs. 20 mg, MD −15.33, 95% from CI −22.84 to −7.829, *p* < 0.001; Figure 3C). In addition, aggrecan production in the group treated with 20 mg bevacizumab was shown to be even higher than the healthy control group (healthy vs. 20 mg, MD −11, 95% CI from −18.5 to −3.495, *p* < 0.001). No significant differences were found among treatment groups (5 vs. 10 mg, MD −1.333, 95% from CI −8.838 to 6.171; 5 vs. 20 mg, MD −6.667, 95% CI from −14.17 to 0.8382; 10 vs. 20 mg, MD −5.333, 95% CI from −12.84 to 2.171). The percentage of MMP-13-positive chondrocytes was significantly lower in the groups injected with bevacizumab compared to the NT group (NT vs. 5 mg, MD 18.93, 95% CI from 10.93 to 26.94, *p* < 0.001; NT vs. 10 mg, MD 24.27, 95% CI from 16.26 to 32.27, *p* < 0.001; NT vs. 20 mg, MD 57.6, 95% CI from 49.59 to 65.61, *p* < 0.001; Figure 3D). Moreover, MMP-13 expression in the group treated with 20 mg bevacizumab was significantly reduced compared to 5 and 10 mg doses (5 vs. 10 mg, MD 5.333, 95% CI from −2.673 to 13.34; 5 vs. 20 mg, MD 38.67, 95% CI from 30.66 to 46.67, *p* < 0.001; 10 vs. 20 mg, MD 33.33, 95% CI from 25.33 to 41.34, *p* < 0.001).

## 4. Discussion

The pathogenesis of OA is complex and still not completely understood. Although classically considered to be a disease of the sole hyaline articular cartilage, novel insights have confirmed that all articular tissues, including the synovium, the subchondral bone, and the joint capsule, contribute to OA pathophysiology [5]. In this scenario, neoangiogenesis of cartilage and synovium has demonstrated a pivotal role in the development of the disease and is primarily initiated by the increased release of VEGF within the joint. VEGF expression in chondrocytes is enhanced by mechanical overloading [2], hypoxia, aging, and in the context of chondrocyte hypertrophy and elevated levels of proinflammatory cytokines, ROS and NO [7]. An increase in VEGF concentration at the osteochondral interface stimulates the process of vascular invasion and tissue reorganization as during endochondral ossification: vessels breach into the avascular cartilage and, subsequently, osteoclasts and chondroclasts are recruited to degrade cartilage ECM, followed by osteoblast-mediated new bone deposition [18]. Indeed, VEGF levels and vascular density have been directly correlated with the degree of chondrocyte hypertrophy [6], chondropathy, and clinical severity of OA [19]. Features of endochondral ossification under VEGF stimulation have been also described during osteophyte formation [20]. Subchondral bone alterations, including sclerosis and cyst formation, have been correlated with increased VEGF concentration as well [10,21]. Another major site of VEGF-mediated neoangiogenesis contributing to OA pathogenesis is the synovium. In osteoarthritic conditions, synovial fibroblasts and macrophages actively secrete VEGF increasing local angiogenesis, which in turn increments inflammation by enhancing cytokine production, vascular permeability, and inflammatory cell infiltration, eventually leading to synovitis [7].

Considering the central role of VEGF in OA pathogenesis, it is reasonable that a specific inhibition of such factors may be efficacious in the etiologic treatment of OA. Bevacizumab is a humanized monoclonal anti-VEGF antibody currently employed to hinder cancer and retinal neoangiogenesis through systemic and intravitreal administration, respectively. Nagai et al. [12] firstly evaluated the effect of systemic vs. intra-articular administration of bevacizumab in an ACLT OA rabbit model. Since the approved dose of bevacizumab in humans is 5 mg/kg but the antibody presents one eighth-fold lower affinity for rabbit VEGF, the intravenous final dose was 40 mg/kg. On the other hand, 25 mg/mL was injected intra-articularly, according to the standard concentration administered intravitreally. At the end of the experimental course, animals from the two groups were treated with a total dose of either 200 mg systemic or 100 mg intra-articular bevacizumab, both showing reduced evidence of articular degeneration, osteophyte formation, and synovial inflammation.

In our study, we performed a dose-response evaluation of three different dosages of intra-articular bevacizumab in a rabbit OA model. In this study, 5, 10, and 20 mg of the antibody were administered once a week for 4 weeks, for a cumulative dose of 20, 40, and 80 mg bevacizumab at the end of the experiment, respectively. We decided to exclude the intravenous route as bevacizumab may be accompanied by serious adverse effects (including cardiac and cerebral ischemia, hypertension, bleeding, and venous thromboembolism) when administered systemically [22]. Consistently with previous reports [12,13], intra-articular administration of bevacizumab was found to be safe, and no measurable reaction or symptom in treated animals was noticed in this study. All three groups treated with the antibody showed a significant reduction of articular cartilage degeneration and synovial inflammation, as demonstrated by the OARSI scores. Furthermore, bevacizumab significantly increased aggrecan and type 2 collagen expression, while decreasing MMP-13 levels, as shown by immunohistochemistry. This was consistent with the results reported by Nagai [12] and Li [13], who both recognized the anticatabolic properties of the antibody. While no major difference was encountered between the groups treated with 5 or 10 mg, animals injected with 20 mg bevacizumab showed better results compared to minor doses with regard to synovial OA changes as well as type 2 collagen and MMP-13 expression. Surprisingly, aggrecan levels in the group treated with 20 mg bevacizumab were found to be higher than the control healthy group. Thus, apart from reducing tissue catabolism and inflammation within the cartilage and synovium, VEGF inhibition may intrinsically have an anabolic effect, hence stimulating the synthesis of ECM components. This mechanism was also proposed by Centola et al. [23], who evaluated a fibrin/hyaluronan scaffold seeded with nasal chondrocytes and subsequently functionalized with bevacizumab both in vitro and in vivo. The presence of the antibody in the construct demonstrated to effectively inhibit vessel ingrowth, support hyaline cartilage production, and increase scaffold survival up to six weeks.

The anabolic effect of bevacizumab may be partially explained by the increased expression of chondromodulin-1 (Chm-1) under VEGF inhibition [12]. Chm-1 is a potent antiangiogenic factor with the role of maintaining avascularity in different tissues, including the inner meniscus, the avascular zone of cartilage and heart valves. It acts by inhibiting endothelial cell-mediated tubule formation and promoting chondrocyte proliferation and differentiation, with an important role in regulating endochondral ossification [24]. Decreased levels of Chm-1 have been reported in OA and appear to be associated with both disease onset and progression [25]. Thus, the loss of Chm-1 during OA favors VEGF-mediated vascular invasion and has been shown to increase chondrocyte hypertrophy, apoptosis, and cartilage ECM degradation via upregulation of hypoxia-inducible factor-2α (HIF-2α) [26]. Intra-articular injection of a lentivirus overexpressing the Chm-1 gene led to a delayed progression of OA in a mouse model, as well as a reduction of VEGF, type X collagen, MMP-13, HIF-2α and TNF-α expression levels [26].

This study has some limitations. Type 2 collagen, aggrecan, and MMP-13 should have been analyzed both at the gene and protein level, as immunohistochemistry only consents a semiquantitative evaluation of target expression. Moreover, VEGF and other OA markers including Chm-1 should have been investigated to better clarify the degree of bevacizumab-mediated VEGF inhibition and its effects on cartilage metabolism. In addition, serum levels of bevacizumab should have been evaluated after the single injection and at the end of the experimental course in order to confirm the safety of the treatment both in the short and long term.

## 5. Conclusions

In the present study, the therapeutic potential of intra-articular bevacizumab was investigated in a rabbit model of OA adopting a dose-response experimental design. The antibody was efficacious in inhibiting cartilage degeneration and synovial alterations typical of the disease at each dosage, with 20 mg being the most effective in terms of aggrecan and type 2 collagen production and MMP-13 inhibition. Because no effective treatment is currently available for OA, the individuation of specific molecular targets responsible for cartilage degeneration is strongly encouraged to develop a targeted therapy. By inhibiting cartilage and synovial neoangiogenesis, bevacizumab holds a promising role in this scenario as a possible disease-modifying osteoarthritis drug (DMOAD). Further investigations to confirm the safety of intra-articular administration of the antibody are encouraged to promote the translation to the clinical setting.

## Data Availability

The data presented in this study are available on request from the corresponding author.
